# Characterization of a New *Providencia* sp. Strain X1 Producing Multiple Xylanases on Wheat Bran

**DOI:** 10.1155/2013/386769

**Published:** 2013-11-20

**Authors:** Abhay Raj, Sharad Kumar, Sudheer Kumar Singh, Mahadeo Kumar

**Affiliations:** ^1^Environmental Microbiology Section, CSIR-Indian Institute of Toxicology Research, M.G. Marg, Post Box No. 80, Lucknow, Uttar Pradesh 226 001, India; ^2^Microbiology Division, CSIR-Central Drug Research Institute, Jankipuram Extension Sector 10, Sitapur Road, Lucknow, Uttar Pradesh 226 003, India; ^3^Animal Facility, CSIR-Indian Institute of Toxicology Research, M.G. Marg, Post Box No. 80, Lucknow, Uttar Pradesh 226 001, India

## Abstract

*Providencia* sp. strain X1 showing the highest xylanase activity among six bacterial isolates was isolated from saw-dust decomposing site. Strain X1 produced cellulase-free extracellular xylanase, which was higher in wheat bran medium than in xylan medium, when cultivated at pH 8.0 and 35°C. Zymogram analysis of crude preparation of enzymes obtained while growing on wheat bran and birchwood xylan revealed the presence of seven and two distinct xylanases with estimated molecular weight of 33; 35; 40; 48; 60; 75; and 95 kDa and 33 and 44 kDa, respectively. The crude xylanases were produced on wheat bran medium and showed optimum activity at pH 9.0 and 60°C. The thermotolerance studies showed activity retention of 100% and 85% at 40°C and 60°C after 30 min preincubation at pH 9.0. It was tolerant to lignin, ferulic acid, syringic acid, and guaiacol and retained 90% activity after ethanol treatment. The enzyme preparation was also tolerant to methanol and acetone and showed good activity retention in the presence of metal ions such as Fe^2+^, Mg^2+^, Zn^2+^, and Ca^2+^. The crude enzyme preparation was classified as endoxylanase based on the product pattern of xylan hydrolysis. Pretreatment of kraft pulp with crude xylanases for 3 h at 60°C led to a decrease in kappa number by 28.5%. The properties of present xylanases make them potentially useful for industrial applications.

## 1. Introduction

Xylan is the major component of the hemicellulosic complex found in the plant cell wall. Complete degradation of xylan requires a coordinated action of several hydrolytic enzymes, among which xylanases (E.C. 3.2.1.8) are sufficient to hydrolyze *β*-1,4-D-xylosidic linkages in xylan. The xylan is the most abundant renewable polysaccharide after cellulose and its hydrolysis by xylanases into simple compounds, mainly xylose, is important for several biotechnological applications. The global demand of xylanase has increased over the years due to its potential application in various industrial processes such as in animal feed, food processing, textiles, pharmacy, and pulp and paper industry [1]. Xylanase is produced by a variety of microorganisms including bacteria, fungi, and actinomycetes [2–4]. However, compared to fungi, major attention has been given to bacteria specially *Bacillus *spp. due to their ability to produce cellulase-free, thermostable and alkaline xylanase [1, 5].

Cellulase-free xylanases functioning at high temperature and pH are required for application in pulp and paper industry, where they reduce toxic wastes, making the bleaching process environmentally friendly. However, relatively high cost of enzyme production has hindered the industrial application of the enzymatic process. In microbial-based enzyme production processes, approximately 30–40% of production of many industries is attributed to the cost of the growth substrates [6]. Xylanases are generally produced when microorganisms are grown in culture media containing xylan and xylan hydrolysate as carbon sources. However, the commercially available xylan is too expensive for use at industrial scale production. Therefore, agroresidues such as wheat bran, rice bran, rice straw, corn stalk, corn cobs, and sugarcane bagasse have been explored as substrates for the production of xylanase. The wheat bran has been found to be the best substrate for higher xylanase production by *Bacillus* spp. [2, 7, 8]. 

Although, a highly purified enzyme is a prerequisite for characterization and structural studies; however, from application perspective, using purified enzyme is not economical for applications like biobleaching [9]. Also, it has been already demonstrated that hydrolysis product profile of crude and purified enzyme preparations are similar [10]. Thus, using agroresidues as substrate for microbial production of xylanase with a combination of all characters, that is, cellulase-free, functions at high pH and temperature could be one of the ways towards substantially reducing the enzyme cost, as verified by recent publications [7, 11]. For this reason, there is still a need of continued search for more efficient xylanolytic bacterial strains that can produce enzymes suitable for pulp biobleaching. In the present study, we screened a new* Providencia *sp. strain X1 from saw-dust decomposing site. It produces multiple xylanases when grown on wheat bran medium. The crude enzyme preparation was alkali- and thermotolerant in nature. 

## 2. Materials and Methods

### 2.1. Chemicals

Chemicals like birchwood xylan, 3,5-dinitrosalicylic acid (DNSA), carboxymethyl cellulose (CMC), Congo red, and D-xylose were purchased from Sigma (St. Louis, MO, USA). Other reagents used in this work were of analytical grade.

### 2.2. Isolation of Xylanolytic Bacteria

Serial dilutions of 10% (w/v) soil sample collected from saw-dust decomposing site, located around a saw mill in Lucknow, Uttar Pradesh, India, were used for screening of the bacteria. Agar plates were made by using modified Berg's mineral salts (BMSs) medium that contained (g/L): NaNO_3_ 3.0, K_2_HPO_4_ 0.5, MgSO_4_·7H_2_O 0.2, MnSO_4_·H_2_O 0.02, FeSO_4_·H_2_O 0.02, and CaCl_2_·2H_2_O 0.02, agar powder 16.0 and yeast extract 5.0 [12], adjusted to pH 7.0 by using 2.0% Na_2_CO_3_, and 1.0% birchwood xylan (w/v) as a source of carbon. After incubation at 35°C for 48 h, six bacterial colonies of distinct morphology (named as X1-X6) were isolated, and their purity was verified by microscopic observation after Gram-staining. 

### 2.3. Screening of Xylanase Producing Isolates

All isolated xylanolytic bacteria were further tested for their xylanase activity by growing them on agar plate and in broth medium. All six isolates were grown on BMS agar plate which contained birchwood xylan (1.0%, w/v). After incubation for 48 h at 35°C, xylan hydrolysis zone was measured by Congo red staining method [13]. These bacterial isolates were rescreened by growing in BMS broth that contained birchwood xylan (1.0%, w/v) at 35°C for 48 h at 120 rpm. The xylanase activity in culture supernatant was measured by using the DNSA method [14]. 

### 2.4. Characterization of Bacterium

The selected bacterium was characterized by conventional and molecular approaches. Morphological and biochemical tests were performed according to standard method [15]. Physiological tests namely, thermotolerance and halotolerance assays were carried out on Petri dishes. For thermotolerance assay, 100 *μ*L culture broths were added to 9.0 mL distilled water and the dilution tubes were heated at 80, 90 and 100°C in water bath for 30 min. Samples (50 *μ*L) were spread onto nutrient agar plates and incubated at 35°C for 48 h. Similarly halotolerance was assayed by adding 8.0% NaCl (w/v) to the media. Molecular characterisation of bacterium was done by 16S rRNA gene sequencing. Total DNA was isolated from bacterium using kit (UltraClean Microbial DNA isolation Kit, MO BIO, USA). The 16S rRNA gene of DNA sample was PCR amplified using 16S rRNA universal primers: 27F (5′-AGAGTTTGATCCTGGCTCAG-3′) and 1492R (5′-TACGGTTACCTTGTTACGACTT-3′) at annealing temperature of 56°C (35 cycles). The PCR product was purified by gel extraction (Gel extraction Kit, Qiagen) and was sequenced in an ABI 3130 genetic analyzer using Big Dye Terminator version 3.1 cycle sequencing kit. The task of sequencing was outsourced to M/s. Eurofins Genomic, a professional company in Bangalore, India. The 16S rRNA gene sequences were compared with available sequences using NCBI-BLAST. A phylogenetic tree was constructed using Bio Informatics Bacterial Identification (BIBI) software (http://umr5558-sud-str1.univ-lyon1.fr/lebibi/lebibi.cgi).

### 2.5. Xylanase Production by Bacterium

Xylanase was produced by selected bacterium in liquid culture condition using BMS broth containing wheat bran and birchwood xylan as production substrate. Erlenmeyer conical flasks (250 mL) containing 100 mL of BMS broth (pH 8.0) supplemented with 1.0% (w/v) of wheat bran or xylan were autoclaved for 20 min at 121°C. The pH of the medium was adjusted using 2.0% Na_2_CO_3_. Flasks were inoculated with 1.0 mL of overnight grown pre-culture with an inoculum size of 1 × 10^6^ CFU/mL on nutrient agar plate and incubated at 35°C, 120 rpm for 120 h (Innova, New Brunswick, USA). The culture broths were withdrawn every 24 h and measured for change of bacterial growth and enzyme activity.

### 2.6. Measurement of Bacterial Growth

Culture broth (2 mL) was withdrawn to measure bacterial growth by taking absorbance at 620 nm in a glass cuvette with 1.0 cm path length (UV-visible 2300 spectrophotometer, Techcomp, Korea). 

### 2.7. Estimation of Enzyme Activity

The culture broth was centrifuged at 10,000 ×g for 30 min and xylanase activity in culture supernatant was determined by measuring the released reducing sugars formed by enzymatic hydrolysis of birchwood xylan. Briefly, 0.5 mL of supernatant and 1.5 mL of 1.0% (w/v) birchwood xylan prepared in 100 mM phosphate buffer, pH 8.0, was incubated at 50°C for 15 min. The reaction was terminated by adding 2.0 mL of 3,5-dinitrosalicylic acid (DNS) reagent. The color development due to formation of reducing sugars was monitored at 540 nm [14], and quantified by comparison with the standard graph of D-xylose. One unit (IU) of xylanase activity was defined as the amount of enzyme that liberated 1 *μ*M of reducing sugars equivalent to D-xylose per minute under the assay conditions. Cellulase activity was assayed by the same method except that low viscosity carboxymethyl cellulose (CMC) was used as substrate. One unit of cellulase activity was defined as the quantity of enzyme releasing 1 *μ*M reducing sugar equivalent to glucose per minute under the assay conditions. 

### 2.8. Zymogram Analysis

Crude enzyme preparations obtained after ammonium sulfate precipitation (80% saturation) and ultrafiltration (Amicon Ultra-15 10 kDa, Millipore) were used for zymogram analysis [16]. Samples (25–30 *μ*g protein) were subjected to SDS-PAGE [17] using 10% polyacrylamide in gel. Electrophoresis was carried out using Mini-Gel Electrophoresis unit (Microkin, Techno Source, Mumbai, India). The samples were loaded in duplicate without the addition of *β*-mercaptoethanol, or any other reducing agents and without boiling to ensure that all active isoforms present there may be detected. After electrophoresis, the gel was cut in two parts. One part was stained with Coomassie brilliant blue R-250 staining and the other portion was used for zymogram analysis. The gel for zymogram analysis was washed two times for 30 min at 4°C in 100 mM sodium phosphate buffer (pH 7.0) containing 25% isopropanol to remove SDS and renature the protein. The gel was then incubated in the same buffer containing 1.0% birchwood xylan solution at 37°C for 30 min to allow the enzyme to digest xylan. Zymogram was developed by staining the gels in Congo red solution (0.1%, w/v) for 15 min. The gel was then destained with NaCl solution (1 M) until zones of clearance and then immersed in 0.5% (w/v) acetic acid to stop the reaction. Presence of halos around the protein bands was considered as enzyme activity. The molecular weight of proteins was determined by comparing them with standard protein marker (GenScript, USA). Total protein concentration in crude xylanase preparations was measured by the method of Bradford [18] using the Genei Protein Estimation Kit (Bangalore Genei, India). 

### 2.9. Partial Characterization of Crude Xylanase Preparation

#### 2.9.1. Optimum pH and Stability of the Xylanase

The optimum pH for xylanase activity was determined by incubating the crude enzyme preparation with 1.0% (w/v) birchwood xylan prepared in 100 mM of citrate buffer (pH 4.0–6.0), phosphate buffer (pH 6.0–8.0), Tris-HCl buffer (pH 8.0–9.0), or glycine-NaOH buffer (pH 9.0–11.0) at 50°C for 15 min. To determine the stability of enzyme at different pH, the crude xylanase preparation was preincubated in the above mentioned buffers of pH 4.0–11.0 at 37°C for 30 min without substrate, and the remaining activity was measured in phosphate buffer (pH 8.0) at 50°C. 

#### 2.9.2. Optimum Temperature and Stability of the Xylanase

The optimum temperature for xylanase activity was determined by incubating the crude enzyme preparation with 1.0% (w/v) birchwood xylan prepared in Tris-HCl buffer (pH 9.0) for 15 min at 30°C, 40°C, 50°C, 60°C, 70°C, 80°C, 90°C, and 100°C. Thermostability of the xylanase was determined by preincubating the crude enzyme preparation at the above mentioned temperatures in Tris-HCl buffer (pH 9.0) without substrate for 30 min, and then residual activity was measured at 60°C. 

#### 2.9.3. Effect of Metals and Solvents on Xylanase Activity

The effect of metal ions on crude xylanase activity was determined by addition of metal ions (FeSO_4_, MnSO_4_, MgCl_2_, ZnSO_4_, CuSO_4_, and CaCl_2_) at 2 mM final concentration in a 2.0 mL reaction mixture. Similarly, the effect of organic solvents on enzyme activity was determined using ethanol, acetone, and methanol at 25% (v/v) concentration in a 2.0 mL reaction mixture. The activity of enzyme without any metal and solvent was considered as control and was taken as 100%.

#### 2.9.4. Effect of Lignin and Its Degradation Product on Xylanase Activity

To determine the effect of lignin and its degradations products on crude xylanase activity, the enzyme assays were performed by the addition of lignin, ferulic acid, gallic acid, syringic acid, guaiacol, and phenol. The effect of lignin was measured over a range of concentration from 0.125 to 1.5 mg/L and the effects of ferulic acid, gallic acid, syringic acid, guaiacol, and phenol were investigated over a range from 2.5 to 10 mg/mL. The enzyme reaction mixture in absence of lignin, ferulic acid, gallic acid, syringic acid, guaiacol, and phenol was treated as a control. All the experiments were carried out in triplicate and the results presented are mean ± SD.

#### 2.9.5. Determination of Exo/Endoxylanolytic Activity

The exo/endoxylanolytic activity of the crude enzyme preparation of strain X1 was examined by carrying out thin layer chromatography (TLC) on silica gel plates. The crude xylanase preparation, control (standard xylan), and treated xylan (1 mL of 1% xylan incubated with 0.5 mL of crude xylanase for 30 min at 50°C) were extracted with methanol [19]. Aliquots of 10 *μ*L for standard D-xylose (1 mg/mL) and methanolic extract of crude xylanase preparation, control, and treated xylan were applied on TLC plate and developed in a solvent mix consisting of *n*-butanol-acetic acid-water (6 : 4 : 3 v/v/v). The TLC plate was sprayed with 99.5% ethanol-concentrated sulfuric acid mix (1 : 1 v/v) and visualized after heating at 100°C.

#### 2.9.6. Biobleaching Studies

One gram unbleached hardwood Kraft pulp (Star Paper Mill, Saharanpur, Uttar Pradesh, India), was washed extensively and treated with 25 mL of crude xylanase preparation (10 IU/mL) at 60°C (pH 9.0) for 3 h at 100 rpm. At the end of reaction, control and enzyme treated pulp samples were filtered, washed with tap water, and dried in an oven at 50°C to a constant weight. Reducing sugars released from untreated and enzyme-treated pulp were measured according to Miller [14]. Kappa number, a measurement of lignin content in pulp, was determined by reaction of pulp with acidified potassium permanganate [20]. All the experiments were carried out in triplicate and the results presented are mean ± SD of the three values.

## 3. Results and Discussion

### 3.1. Isolation and Characterization of Bacterial Strain X1

**Figure 1:**
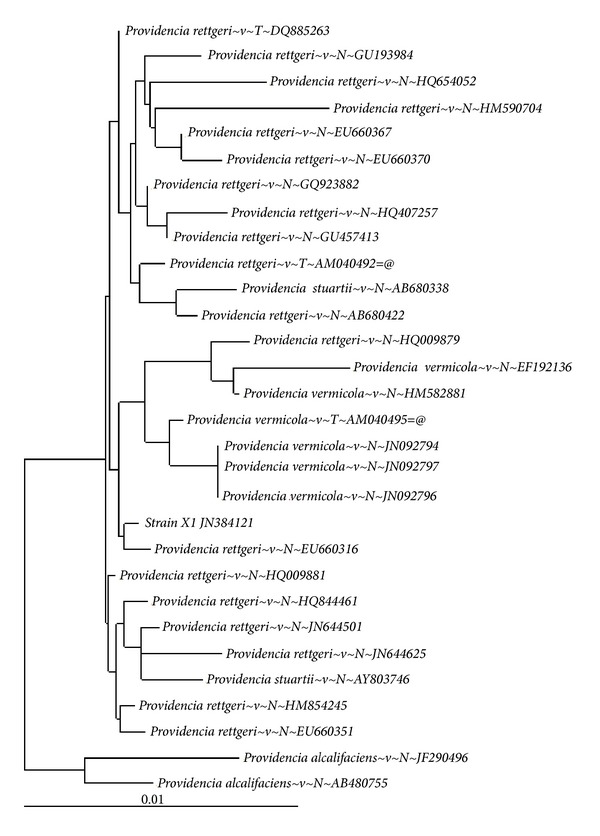
Phylogenetic tree of the *Providencia *sp. strain X1 and their related genera has been linked based on 16S rRNA sequence comparisons.

Six bacterial strains (X1-X6) which are morphologically distinct and formed clear zones on xylan-agar plate were isolated from saw-dust decomposing soil. Strain X1 which showed the highest clearance was selected for further characterization. The property was corroborated by measurement of xylanase activity, which again was the highest (17.4 ± 0.4 IU/mL) in strain X1 (Table 1). The extracellular enzyme matrix from strain X1 was free from cellulase contamination (data not shown). Strain X1 was Gram-positive and spore forming rod. Also, it was catalase, oxidase, caseinase, and tryptophanase positive. It was able to metabolize variety of sugars except fructose and sucrose (Table 2). The isolate was moderately thermophilic and thermotolerant. It grew at 50°C and showed its tolerance up to 90°C for 30 min. It was also tolerant to alkaline pH (pH 10) and salt stress (NaCl 8.0%). This ability to withstand temperature, salt stress, and pH stress may be an intrinsic property of the strain [21]. The nucleotide blast analysis of 16S rDNA sequence showed >98% identity with several *Providencia *sp.; however, maximum homology (99%) was observed with *Providencia rettgeri* (Figure 1). The 16S rDNA sequence has been deposited in NCBI GenBank (Accession no. JN384121). 

### 3.2. Xylanase Production by Strain X1

**Figure 2:**
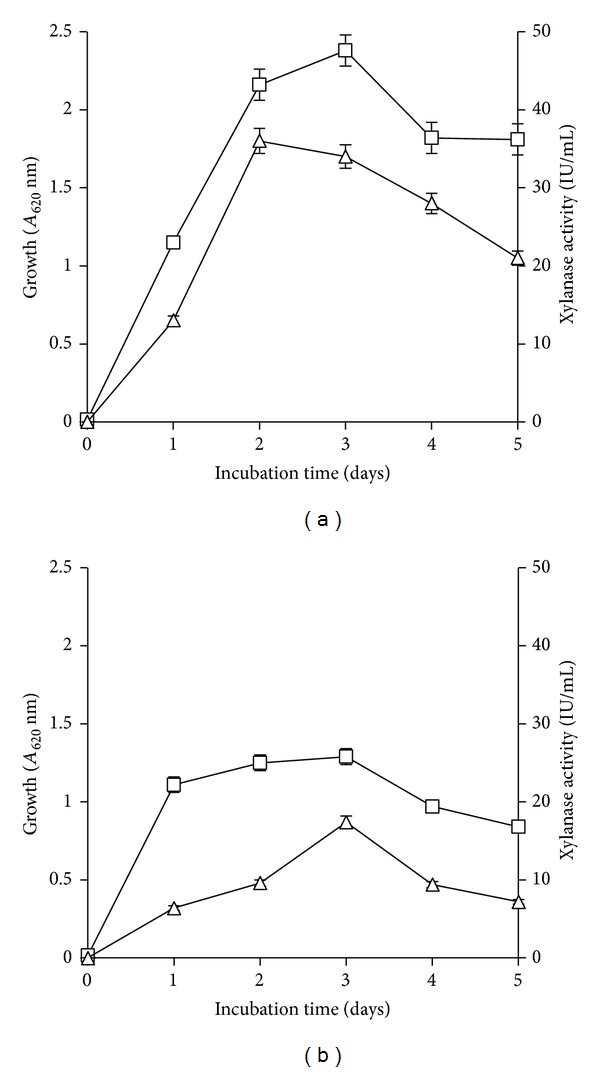
Growth (□) and xylanase (△) production by strain X1 in wheat bran (a) and xylan medium (b) at 35°C, pH 8.0 and shaking at 120 rpm.

The xylanase production studies suggested that wheat bran as substrate was suitable for growth and enzyme production. The bacterial strain when grown on wheat bran produced extracellular xylanase (36.3 ± 1.6 IU/mL) after 48 h of incubation (Figure 2(a)). The enzyme production was lower when grown on pure xylan (17.4 ± 0.8 IU/mL) after 48 h of incubation and a declining trend was observed afterwards (Figure 2(b)). Higher xylanase activity in wheat bran medium could be due to improved bacterial growth (OD_620_ 2.4) in this medium, compared to (OD_620_ 1.3) in xylan medium at 48 h (Figures 2(a) and 2(b)). High production of xylanase in wheat bran medium has also been reported earlier during submerged fermentation (SmF) of *Geobacillus thermoleovorans* and *Bacillus pumilus* strains MK001 [22, 23]. The suitability of wheat bran as substrate could be due to the presence of 45% hemicellulose, which may fulfill the role of inducer and 23% organic nitrogen which is essential for protein synthesis [24]. 

### 3.3. Zymogram Analysis

**Figure 3:**
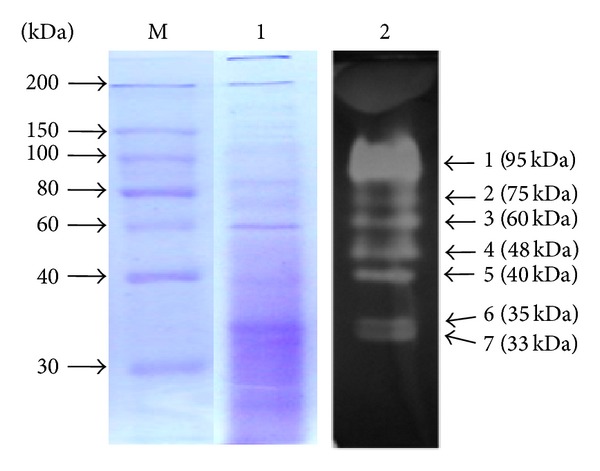
Zymogram analysis shows presence of seven distinct xylanases in crude enzyme preparation of strain X1 grown on wheat bran. Lane 1: Coomassie brilliant blue stained gel; Lane 2: Congo red followed by acetic acid stained gel; and Lane M: protein molecular weight marker.

**Figure 4:**
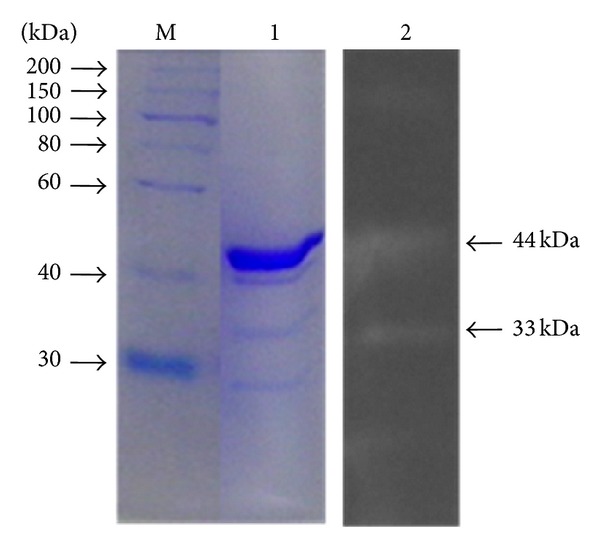
Zymogram analysis shows presence of two xylanases in crude enzyme preparation of strain X1 grown on birchwood xylan. Lane 1: Coomassie brilliant blue stained gel; Lane 2: Congo red followed by acetic acid stained gel; and Lane M: protein molecular weight marker.

Zymogram technique was employed to observe the presence of multiple forms of xylanase expressed in presence of wheat bran and birchwood xylan. The zymogram analysis of crude xylanase preparation of organism grown on wheat bran contained multiple xylanases as indicated by the presence of halos around seven distinct proteins bands corresponded to estimated molecular weight of 33, 35, 40, 48, 60, 75, and 95 kDa (Figure 3). In contrast, crude xylanase preparation of organism grown on birchwood xylan contained only two xylanases of 33 kDa and 44 kDa (Figure 4). This suggested differential expression of xylanases in *Providencia *sp. strain X1 as has also been reported earlier from *Myceliophthora *sp. [25]. Multiplicity of xylanases has been documented in various microorganisms with evidence for the occurrence of 3 to 30 xylanases in bacteria and fungi [6], possibly as a result of differential mRNA processing and posttranslational modifications, as well as the product of multiple genes [26, 27]. To the best of our knowledge, this is the first evidence of large number of xylanases induction in bacteria by wheat bran. The presence of multiple xylanases in crude preparation from one organism could synergistically function for superior xylan hydrolysis [21]. 

### 3.4. Characterization of Crude Xylanase Preparation of Organism Grown on Wheat Bran

#### 3.4.1. Effects of pH and Temperature on Activity and Stability of Xylanase

**Figure 5:**
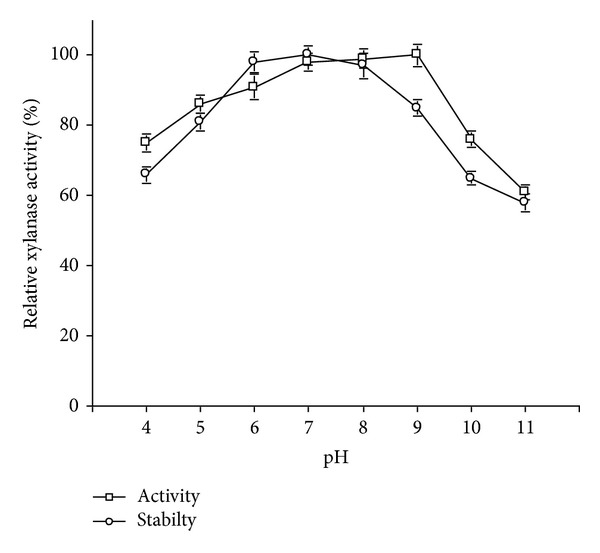
Effect of pH on activity and stability of crude xylanase preparation of strain X1.

The crude xylanase preparation was active in the pH range of pH 4.0–11.0 (>60% activity) with the highest activity at pH 9.0 (Figure 5). Also, the enzyme activity was 76% and 61% of its optimum activity, at pH 10.0 and pH 11.0, respectively. The stability studies at different pH suggested that crude enzyme preparation was stable over the pH ranges of 4.0–11.0, but retained 97–100% of its residual activity after 30 min preincubation at pH 6.0–pH 8.0 (Figure 5). However, more than 80%, 65%, and 58% of its activity was retained by the enzyme after preincubation at pH 9.0, pH 10, and pH 11.0 respectively, thus suggesting that crude enzyme preparation was moderately stable at elevated pH. Most of the xylanases isolated from the *Bacillus *spp. exhibited the highest activity within pH range of 6.0–8.0 [28–31]. Although, xylanases isolated from *Bacillus *sp. strain 41 M-1 and *Bacillus* sp. AR-009 [32, 33] showed the highest activity at pH 9.0, but they have lower stability at this pH values. At pH 9.0, xylanase isolated from *Bacillus *sp. AR-009 retained only 54% of its activity after 1 h [33]. 

**Figure 6:**
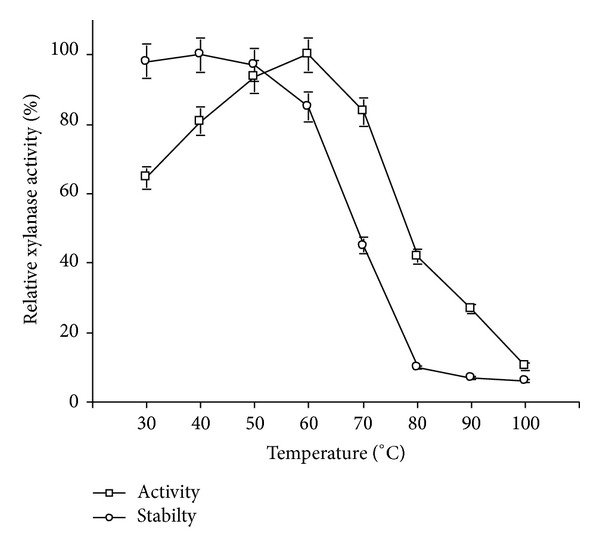
Effect of temperature on activity and stability of crude xylanase preparation of strain X1.

The activity of crude xylanase preparation was the highest at 60°C. The enzyme retained more than 80% and 40% of the maximum activity when assayed at 70°C and 80°C, respectively (Figure 6). The stability studies at different temperatures revealed that crude xylanase preparation did not lose significant activity up to 50°C after 30 min preincubation. However, it retained 85% and 45% of its original activity after 30 min incubation at 60°C and 70°C, respectively. Above 70°C, the enzyme's stability decreased gradually and only 10% activity was retained at 80°C (Figure 6). The significant enzyme stability at higher temperatures, that is, 50°C and 60°C, would be important for its industrial application. Most of the xylanases isolated from *Bacillus *spp. showed the highest activity in the temperature range of 50–55°C [28, 30, 34]. Some xylanases of bacterial origin showed optimum activity at 60°C, but their pH optima were less than pH 9.0 [35, 36]. Differently, in the present study, the optimum pH and temperature for xylanase were 9.0 and 60°C, respectively, thereby suggesting it to be an alkali and thermotolerant enzyme. 

#### 3.4.2. Effect of Metal Ions and Solvents on Enzyme Activity

The addition of Fe^2+^, Mg^2+^, Zn^2+^, and Ca^2+^ ions at final concentration of 2 mM showed a partial inhibition on activity of the crude xylanase (retaining 93–98% of activity), whereas addition of 2 mM of Mn^2+^ strongly inhibited the xylanase activity by 65% (Table 3). Mn^2+^ has earlier been reported to strongly inhibit the activity of the xylanase of *Bacillus halodurans* S7 [37]. In contrast to this study, presence of Fe^2+^, Mg^2+^, Zn^2+^, and Ca^2+^ ions at the concentration of 1 mM increased 63%, 56%, 63%, and 31% activity of xylanase from *Arthrobacter *sp. MTCC 5214, receptively [38]. The stability studies with hydrophilic solvents suggested it to be showing good solvent tolerance in 25% ethanol (91%), methanol (82%), and acetone (78%) (Table 3). In contrast, complete inhibition of *Macrotermes subhyalinus* xylanase was observed at 30% and 60% v/v of alcohols (methanol, ethanol, propanol, and butanol) [39]. Impurities like metal ions and solvents, which can potentially inhibit the activity of xylanase, exist in raw pulp. Thus, significant activity of xylanase isolated from *Providencia *sp. strain X1 in presence of metal ions (Fe^2+^, Mg^2+^, Zn^2+^, and Ca^2+^) and hydrophilic solvents enhances its suitability for paper pulping applications. 

#### 3.4.3. Effect of Lignin and Phenolic Compounds on Activity of Xylanase

**Figure 7:**
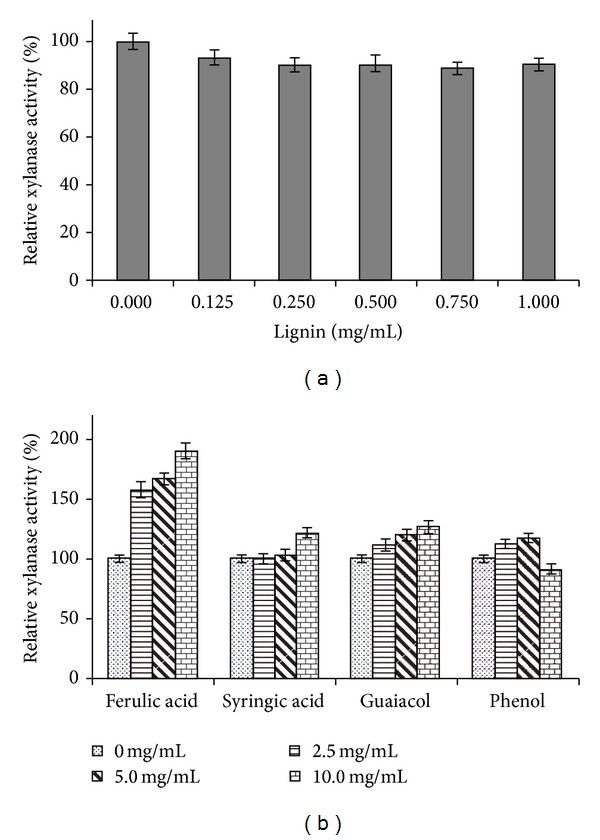
Influence of lignin (a) and its monomeric compounds (b) on activity of crude xylanase preparation of strain X1.

The effect of soluble lignin on xylanase activity was investigated at lignin concentration ranging from 0 to 1.5 mg/mL. The result showed that inhibition in xylanase activity was observed at low lignin concentration with a 10% reduction in activity at 0.25 mg/mL of lignin. However, no increase in inhibition was observed at higher concentrations of lignin (Figure 7(a)). The inhibition in enzyme activity can be correlated with either precipitation of protein out of the solution or by binding of the enzyme onto lignin [40]. Morrison et al. [41] have studied the impact of soluble lignin and found a 25% inhibition in enzyme activity at low concentration of lignin (0.075 mg/mL). In contrast, Kaya et al. [42] reported that the addition of lignin at increasing concentrations (0–0.06%) increased the hydrolysis rate of xylanase by more than 20%. Berlin et al. [43] examined the inhibition of cellulase, xylanase, and *β*-glucosidase by lignin preparation. Cellulase displayed varying levels of inhibition while xylanases displayed consistent inhibition and *β*-glucosidase was the least affected. 

The kraft pulp contains trace amount of low molecular weight phenolic compounds, which may potentially affect the activity of enzyme. In the present study, four phenolic compounds, namely, ferulic acid, syringic acid, guaiacol, and phenol, were tested to see their effect on xylanase activity at concentrations ranging from 0 to 10 mg/mL. These compounds are either lignin degradation products or naturally present in plant biomass. Addition of ferulic acid, syringic acid, and guaiacol increased the activity of xylanase by 91%, 21%, and 26%, respectively, as compared to control at concentration of 10 mg/mL (Figure 7(b)). Conversely, phenol had less inhibitory effect on xylanase activity at higher concentration (10 mg/mL). Morrison et al. [41] have examined the impact of *ρ*-coumaric acid and gallic acid on xylanase activity and found 85% and 84% inhibition at low concentration (0.1%) of *ρ*-coumaric acid and gallic acid. Kaya et al. [42] found that at low concentration (0.05%) of vanillin acid, acetovanillone, guaiacol, and protocatechuic acid increased the rate of hydrolysis of xylan. Other authors have also investigated the effect of phenolic compounds and concluded that the degree of inhibition varied depending on the enzyme, the microorganism it was derived from, and the phenolic compound tested [44]. 

#### 3.4.4. Endoxylanolytic Activity

**Figure 8:**
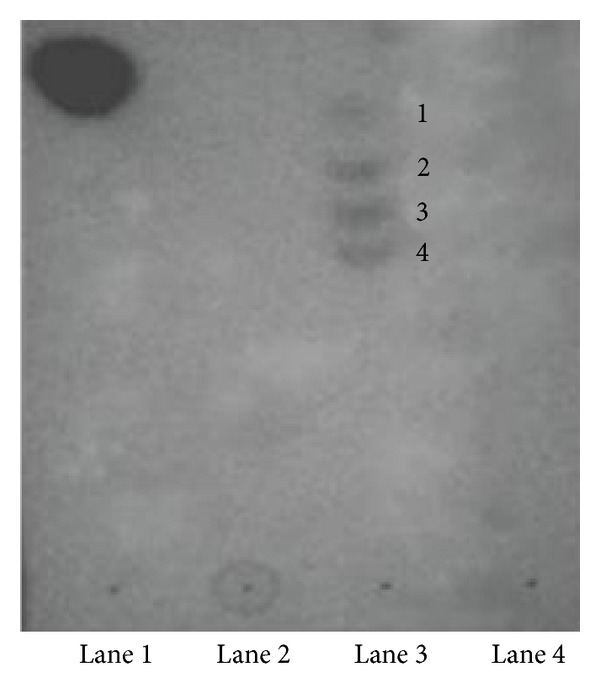
Demonstration of endoxylanolytic nature of crude xylanase preparation of strain X1. Lane 1 indicates standard D-xylose. Lanes 2 and 3 represent the methanol extract of control and treated xylan, while Lane 4 represents crude xylanase, respectively. Numbers (1–4) represent compounds present in crude xylanase-treated xylan.

The thin layer chromatography (TLC) (Figure 8) study revealed absence of bands in methanolic extract of control xylan and crude xylanase (Lanes 2 and 4). The methanolic extract of treated xylan (Lane 3) revealed three bands which were below the standard D-xylose (Lane 1). These results indicated that *Providencia *sp. strain X1 xylanase released a range of xylooligosaccharides from xylan, suggesting it to be an endoxylanase. Similarly, xylanase produced by *Bacillus halodurans *S7 was an endoxylanase [37].

#### 3.4.5. Pulp Pretreatment Studies

**Figure 9:**
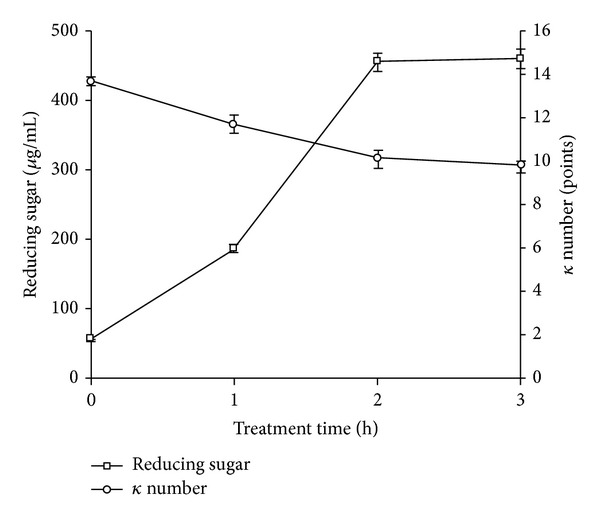
Effect of crude xylanase preparation treatment on level of the reducing sugars released and kappa number of kraft pulp.

The biobleaching efficiency of *Providencia *sp. strain X1 xylanase was studied by treatment of pulp with crude enzyme (10 IU/mL) after incubation of 0 to 3 h. The results reveal gradual increase of reducing sugars with time. The amount of reducing sugars present in filtrate of untreated pulp was 59 *μ*g/mL and it increased to 185, 455, and 460 *μ*g/mL in filtrate of treated pulp after 1, 2, and 3 h, respectively (Figure 9). Kamble and Jadhav [31] reported that the amount of reducing sugars released from kraft pulp by the xylanase isolated from *Cellulosimicrobium* sp. MTCC 10645 was significantly greater with increasing time. Kappa number is the measurement of the amount of lignin present in the pulp. Kappa number of untreated pulp was 13.7. After treating it with enzyme, it decreased to 9.8, which indicates that the kappa number of pulp decreased by 28.5% after 3 h incubation. Biobleaching of hardwood kraft pulp with crude xylanase dose of 20 IU g^−1^ pulp from *Bacillus megaterium* and *B. pumilus *ASH has resulted in a decrease in kappa number by 11 to 13.6% after 3 h treatment at 50°C and 60°C, respectively [7, 11]. While, in the present study, the treatment for 3 h at 60°C using crude xylanases from *Providencia *sp. strain X1 led to 28.5% reduction in kappa number. 

## 4. Conclusions

The pulp and paper industry requires cellulase-free and thermoalkalophilic xylanase that can be produced at low cost. In the present study, the isolated bacterial strain X1 produces xylanases using wheat bran as a substrate. The xylanases produced by present isolate are cellulase-free and are alkali and thermotolerant. They are also tolerant to higher lignin concentrations as well as its degradation products and to metal ions and solvents. The significant reduction in kappa number of pulp by crude xylanase preparation suggested *Providencia *sp. strain X1 to be a potential source of xylanase for pulp biobleaching. 

## Figures and Tables

**Table 1:** Screening test for selecting bacteria with high xylanase producing ability.

Isolate no.	Observation of plate assay^a^			Xylanase activity^b^ (IU/mL)
	Clearance zone diameter (cm)	Colony diameter (cm)	Diameter ratio	
X1	2.5	1.4	1.8	17.4 ± 0.4
X2	1.3	1.3	1.0	9.6 ± 0.2
X3	2.5	2.5	1.0	6.4 ± 0.1
X4	2.4	1.3	1.8	9.4 ± 0.2
X5	1.4	1.4	1.0	4.6 ± 0.1
X6	2.3	2.1	1.1	7.2 ± 0.1

^a^Medium used: Berg's mineral salts-xylan agar; growth conditions: pH 7.0, 35°C, 48 h.

^b^Medium used: Berg's mineral salts-xylan broth; growth conditions: pH 8.0, 35°C, 48 h, 120 rpm.

**Table 2:** Morphological and biochemical characteristics of strain X1.

Test	Results
Whole colony	White, circular
Edge	Entire
Surface	Smooth
Elevation	Raised
Cell shape	Rod
Spore position	Subcentral
Gram-reaction	+
Growth at 50°C	+
Growth at 8.0% NaCl	+
Motility	+
Catalase	+
Oxidase	+
Caseinase	+
Urease	−
Gelatinase	−
Tryptophanase	+
Sugar utilization	
Glucose	+
Fructose	−
Lactose	+
Maltose	+
Xylose	+
Sucrose	−
Arabinose	+
Mannose	+

+: positive; −: negative.

**Table 3:** Effect of metal ions and solvents on activity of crude xylanase preparation from strain X1.

Additive	Relative xylanase activity (%)
None	100 ± 4.0
Metal	
CaCl_2_	98 ± 4.0
FeSO_4_	95 ± 4.5
ZnSO_4_	95 ± 3.2
MgCl_2_	93 ± 4.1
CuSO_4_	78 ± 3.0
MnSO_4_	35 ± 4.4
Solvent	
Ethanol	91 ± 4.3
Methanol	82 ± 3.8
Acetone	78 ± 5.0
